# Variation in Specificity of HIV Rapid Diagnostic Tests over Place and Time: An Analysis of Discordancy Data Using a Bayesian Approach

**DOI:** 10.1371/journal.pone.0081656

**Published:** 2013-11-25

**Authors:** Derryck Klarkowski, Kathryn Glass, Daniel O’Brien, Kamalini Lokuge, Erwan Piriou, Leslie Shanks

**Affiliations:** 1 Médecins sans Frontières, Operational Centre Amsterdam, Amsterdam, The Netherlands; 2 Australian National University, Canberra, Australia; 3 Department of Medicine and Infectious Diseases, Royal Melbourne Hospital, University of Melbourne, Melbourne, Australia; University of Cape Town, South Africa

## Abstract

**Background:**

Recent trends to earlier access to anti-retroviral treatment underline the importance of accurate HIV diagnosis. The WHO HIV testing strategy recommends the use of two or three rapid diagnostic tests (RDTs) combined in an algorithm and assume a population is serologically stable over time. Yet RDTs are prone to cross reactivity which can lead to false positive or discordant results. This paper uses discordancy data from Médecins Sans Frontières (MSF) programmes to test the hypothesis that the specificity of RDTs change over place and time.

**Methods:**

Data was drawn from all MSF test centres in 2007-8 using a parallel testing algorithm. A Bayesian approach was used to derive estimates of disease prevalence, and of test sensitivity and specificity using the software WinBUGS. A comparison of models with different levels of complexity was performed to assess the evidence for changes in test characteristics by location and over time.

**Results:**

106, 035 individuals were included from 51 centres in 10 countries using 7 different RDTs. Discordancy patterns were found to vary by location and time. Model fit statistics confirmed this, with improved fit to the data when test specificity and sensitivity were allowed to vary by centre and over time. Two examples show evidence of variation in specificity between different testing locations within a single country. Finally, within a single test centre, variation in specificity was seen over time with one test becoming more specific and the other less specific.

**Conclusion:**

This analysis demonstrates the variable specificity of multiple HIV RDTs over geographic location and time. This variability suggests that cross reactivity is occurring and indicates a higher than previously appreciated risk of false positive HIV results using the current WHO testing guidelines. Given the significant consequences of false HIV diagnosis, we suggest that current testing and evaluation strategies be reviewed.

## Introduction

The UNAIDS strategy “Getting to Zero” is an ambitious programme to reduce to zero both the number of new HIV infections and AIDS related deaths[1]. Recent evidence that treatment can prevent transmission of disease, along with a gathering consensus that anti-retroviral treatment (ART) should be started earlier during the asymptomatic stage of HIV, underlines the importance of increasing access to HIV testing [2,3]. New WHO guidelines on counseling and testing now discuss self-testing strategies for the first time [4]. These developments increase the need for accurate HIV testing. 

The WHO HIV testing strategy recommends the use of rapid diagnostic tests (RDTs) combined in an algorithm of two or three tests for the diagnosis of HIV in resource limited settings [4]. HIV RDTs are susceptible to cross reactivity with non-HIV antibodies and this can lead to discordant (one test positive, one test negative) and false positive results (both tests falsely positive). 

The CDC/WHO/APHL “Guidelines for Appropriate Evaluation of HIV Testing Technologies in Africa” provide a 3 stage system for development of a diagnostic HIV RDT algorithm [5]. After a review of the RDTs from published data, an evaluation of the RDTs chosen is done using a sample serobank followed by pilot testing in the field and then ongoing monitoring of testing quality. However implementation of these guidelines is generally beyond the capacity of most programmes and in many cases tests are introduced and algorithms formulated without preceding local validation [6]. 

Furthermore the guidelines assume that a population is serologically stable over time, as once adequate test performance is established through the initial validation, no ongoing assessment of test specificity or sensitivity is required. Our experience across more than 22 programmes in 10 countries, suggest that this is not the case. This paper uses discordancy data from Médecins Sans Frontières - Operational Centre Amsterdam (MSF) testing programmes to test the hypothesis that the specificity of RDTs change over time and place. 

## Methods

### Inclusion Criteria

Data was drawn from HIV test centres where MSF offered access to free counselling and testing (CT) services as part of its routine programmes in 2007-8. All centres reported monthly testing volumes, HIV positivity rates, and rates and patterns of discordancy. Test centres were included if an algorithm was used where two independent RDTs were used in parallel for all persons tested, and if at least 3 months of data representing >90 patient/client results was reported. 

### Quality control

Tests were performed according to MSF standard operating procedures (SOPs) based on the manufacturer’s instructions. All rapid test kits were stored according to manufacturers' instructions. Tests were performed by trained counsellors or nurses. Quality control for the test device was performed using a weak-positive sample prepared by the MSF supervising laboratory on each new test kit, and at least once weekly. Quality control for correct execution and interpretation of the test was performed in the laboratory by repeat testing of a random sample of 20 positive and 20 negative samples each month. Invalid test results whereby the control line did not appear were discarded, and repeated on a new test device. As an additional quality control measure, all positive or discordant results were repeated in the laboratory by a technician on a venous sample. Efforts were made to blind the technician to the results of the first tests. Where there was discrepancy between the results of the counsellor/nurse and the laboratory, the test was repeated a third time in the laboratory to determine the final outcome using a test from a different test kit and where possible from a different batch. 

### Analysis

In the absence of reference laboratory testing to assess false positive and false negative results, a Bayesian approach was used to derive estimates of disease prevalence, and of test sensitivity and specificity using the software WinBUGS. This approach allows calculation of test characteristics without a gold standard for comparison by specifying equations linking prevalence, specificity and sensitivity to data [7], [8], [9]. Logistic transforms were used to ensure that the distributions for parameters lay between 0 and 1. Prior distributions for prevalence were uninformative, while sensitivity and specificity parameters were given priors with median value 0.99, and credible interval (0.24, 1.0) to incorporate prior knowledge on the likely values for these test statistics. A comparison of results with uninformative priors gave broadly consistent output, but led to convergence difficulties in some cases. 

There are four possible test results for each individual, namely: both tests positive, test A positive and test B negative, test A negative and test B positive, and both tests negative. For each location, and each month, we estimated disease prevalence (p), sensitivity (v_A_, v_B_) and specificity (f_A_, f_B_) of the two tests using formulae that define the probability of these four test results. For example, the probability of two positive test results is:

Probability (positive,positive) = p*v_A_*v_B_ + (1-p)*(1-f_A_)*(1-f_B_)

and the number of individuals with two positive tests has a binomial distribution with N equal to the total number of individuals tested, and the event probability as above. Since dependencies in the data prevent estimation of all parameters independently, we included a further association between sensitivity and specificity to ensure convergence of the full model. Details of the choice of this assumption, together with a detailed listing of all model equations are provided as Supporting Information (see Annexe S1 and Figure S1).

### Model Selection

To assess the variation in algorithm results across different programmes and over time within the same programme, we compared model fit statistics for a sequence of models with increasing numbers of parameters. All models estimated prevalence at each site independently. The simplest model (Model 0) assumed that all tests have the same sensitivity and specificity. Model 1 allowed for each test to have a different specificity and sensitivity, but assumed this is fixed over locations. Model 2 included variability in test characteristics by location, while Model 3 also included variability over time. We used the Deviance Information Criterion (DIC) within WinBUGS to compare model fit, with smaller values of the DIC indicating better fit. The best-fit model was then used to assess whether variation in specificity over time and place within a single country and /or site was likely to be of value in predicting discordancy rates. 

In order to assess the roles of sensitivity and specificity in discordant results, we used the estimated values of test sensitivity (v_A_ and v_B_) and test specificity (f_A_ and f_B_) in each location – together with the prevalence and the total number of tests – to predict the proportion of discordant results that were due to false positives and false negatives. 

In light of findings that most discordant results were due to false positives, we developed an alternative ‘perfect sensitivity’ model in which we assume all discordant results are due to false positives. That is, we assume v_A_ = v_B_ = 1 at all locations. While not used for any of the main results in this paper, this alternative model allows us to assess the impact of model assumptions on our results.

### Ethics Statement

This analysis met the criteria set by the Médecins Sans Frontières Ethics Review Board, for retrospective analysis of routinely collected programmatic data. Data used in this analysis are aggregate data without personal identifiers and therefore individual patient consent was not obtained. 

## Results

106,035 individuals comprising a total of 212,070 tests, were included from 51 centres in 10 countries. Determine® was used for all testing together with Capillus®, Hexagon®, First Response®, SD Bioline®, Tridot® and Unigold®. A summary of countries, test performed, discordancy rates and patterns of discordancy is given in Table 1. HIV prevalence by test site within each country can be found in the Information (see Figure S2). A break-down of the number of tests done in each test centre is available in the Information (Annexe S2).

**Table 1:** Overview of countries and patients tested.

**Country**	**Second test**	**Patients tested**	**Total discordant**	**Percentage Determine® +** ^1^
CAR	Unigold®	4, 329	143 (3.3%)	92%
Congo-B	Unigold®	438	26 (5.9%)	46%
DRC	Unigold®	19, 065	119 (0.6%)	74%
Ethiopia	Unigold®	4, 961	363 (7.3%)	15%
Haiti	Unigold®	2, 006	20 (1.0%)	95%
	Capillus®	4, 458	54 (1.2%)	100%
India	Unigold®	1, 193	19 (1.6%)	58%
	TriDot®	4, 266	59 (1.4%)	97%
Ivory Coast	Unigold®	3, 386	125 (3.7%)	90%
	Hexagon®	5, 648	334 (5.9%)	39%
Myanmar	Unigold®	14, 796	331 (2.2%)	90%
	Capillus®	25, 024	528 (2.1%)	73%
Uganda	Unigold®	6, 056	129 (2.1%)	81%
Zimbabwe	First Response®	7, 782	18 (0.2%)	67%
	SD Bioline®	2, 627	11 (0.4%)	73%

^1^ Refers to the percentage Determine® test positive amongst the discordant tests.

**Figure 1:**
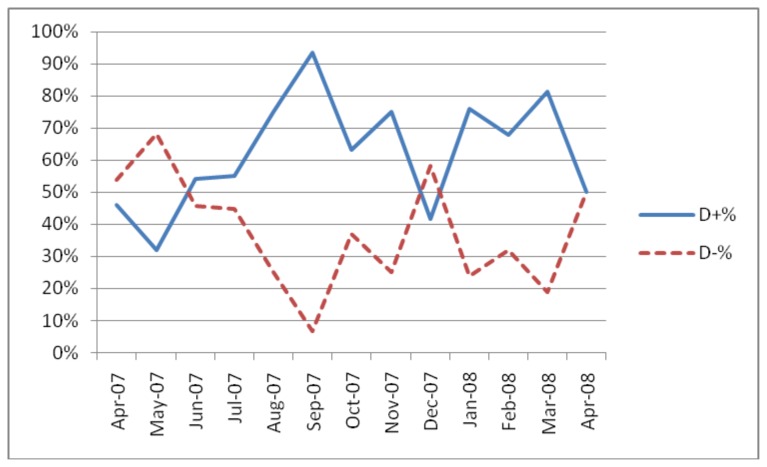
Discordancy pattern in Kachin, Myanmar test centre using Determine® and Capillus®.

**Figure 2:**
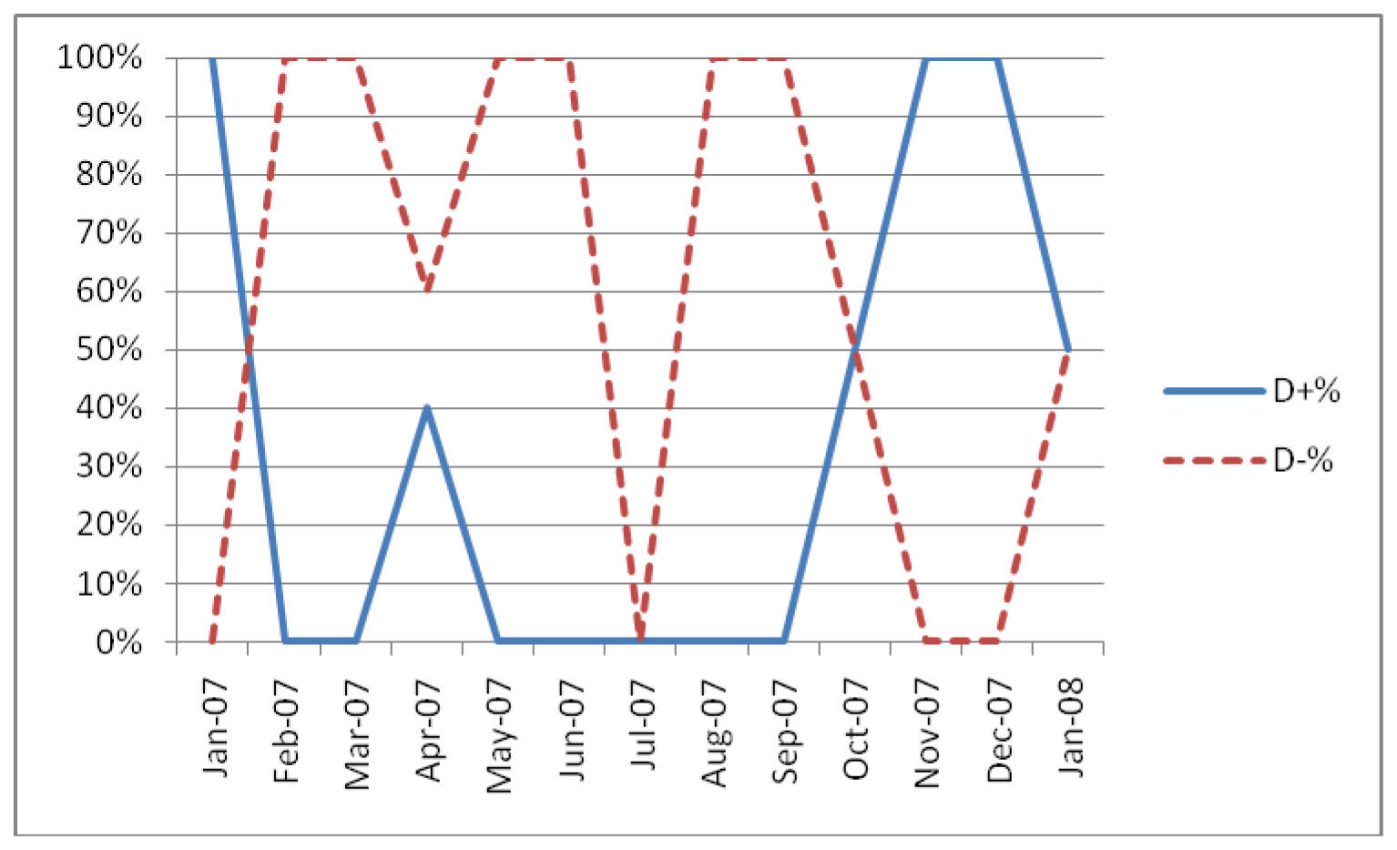
Discordancy pattern in Mindouli, Congo-Brazzaville (Determine®, Unigold®).

Several of the test centres experienced considerable variability in the pattern of discordancy (testA+/testB- versus testA-/testB+) as shown in Figures 1 and 2. 

### Model Comparison

Table 2 describes the sequence of models fit to data together with the Deviance Information Criterion (DIC) statistic used to measure the strength of model fit, with smaller numbers indicating an improvement in fit. The large intervals between the DIC numbers give strong support for the inclusion of additional factors, providing clear evidence of a better fit to the data when variability by place and variability over time are included in the model.

**Table 2:** Sequence of models fitted together with Deviance Information Criteria (DIC).

Model	**Description**	**DIC**
Model 0	All tests have equal sensitivity and specificity.	10, 028
Model 1	Sensitivity and specificity vary by test but not location or time.	9, 183
Model 2	Sensitivity and specificity vary by test and location but not time.	7, 576
Model 3	Sensitivity and specificity vary by test, location and time.	6, 149

This indicates goodness of fit of the model to data, with smaller values indicating a better fit.

### Variation by geographical location

**Figure 3:**
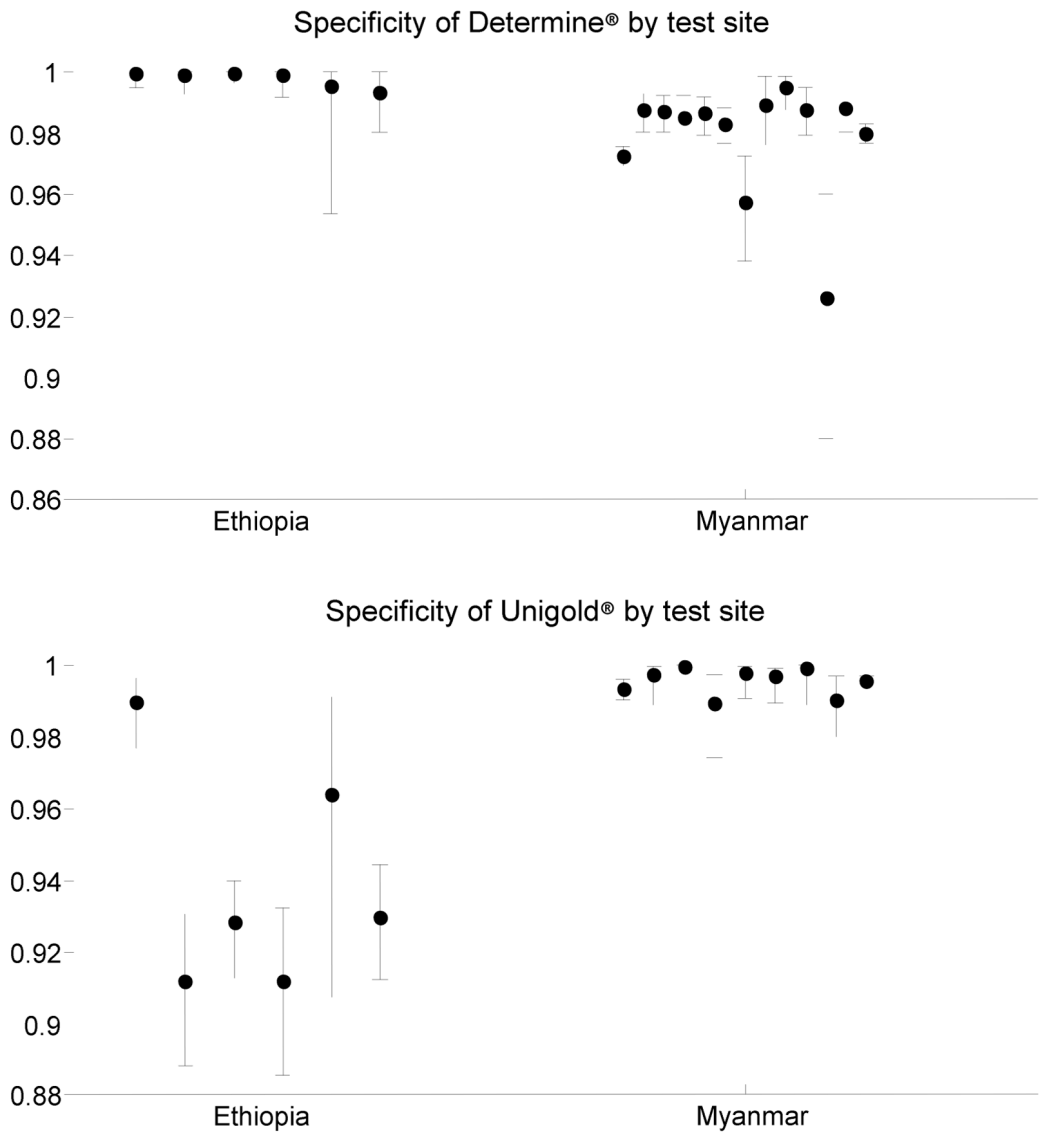
Estimates of specificity of Determine® and Unigold® in Ethiopia and Myanmar. The dots show the point estimate at a test site, and the bars give 95% credible intervals. All estimates are adjusted for site prevalence and test sensitivity. Note that some sites in Myanmar used Capillus® as the second HIV test, so that the number of sites with estimates of Unigold® specificity is fewer than the number of sites with estimates of Determine® specificity. The data included a median of 650 individuals per site (range 101-1,901) in Ethiopia, a median of 1,358 individuals per site (range 193-15,094) tested using Determine® in Myanmar and a median of 569 individuals per site (range 193-7,004) tested using Unigold® in Myanmar.

We show evidence of variation in test characteristics by place using the estimated specificity of Determine® and Unigold® (the most commonly used tests in the dataset) at different sites in two countries: Ethiopia and Myanmar. Figure 3 shows point estimates and 95% credible intervals for the estimates of specificity at each test site in each of 6 test sites in Ethiopia, and 13 sites in Myanmar. By estimating all parameters simultaneously, we have ensured that estimates of specificity are adjusted for disease prevalence and test sensitivity by test site. Determine® was found to perform well in all sites in Ethiopia, but shows variable specificity in Myanmar, with specificity at one site as low as 92.6%. In contrast, Unigold® performs well in Myanmar but poorly in Ethiopia, with point estimates of specificity below 93% at a number of sites. Further examples of variable specificity by location can be found in the Information (see Figure S3).

The data provide evidence of variation in test specificity between different testing locations within a single country. 

### Variation in the same programme over time

**Figure 4:**
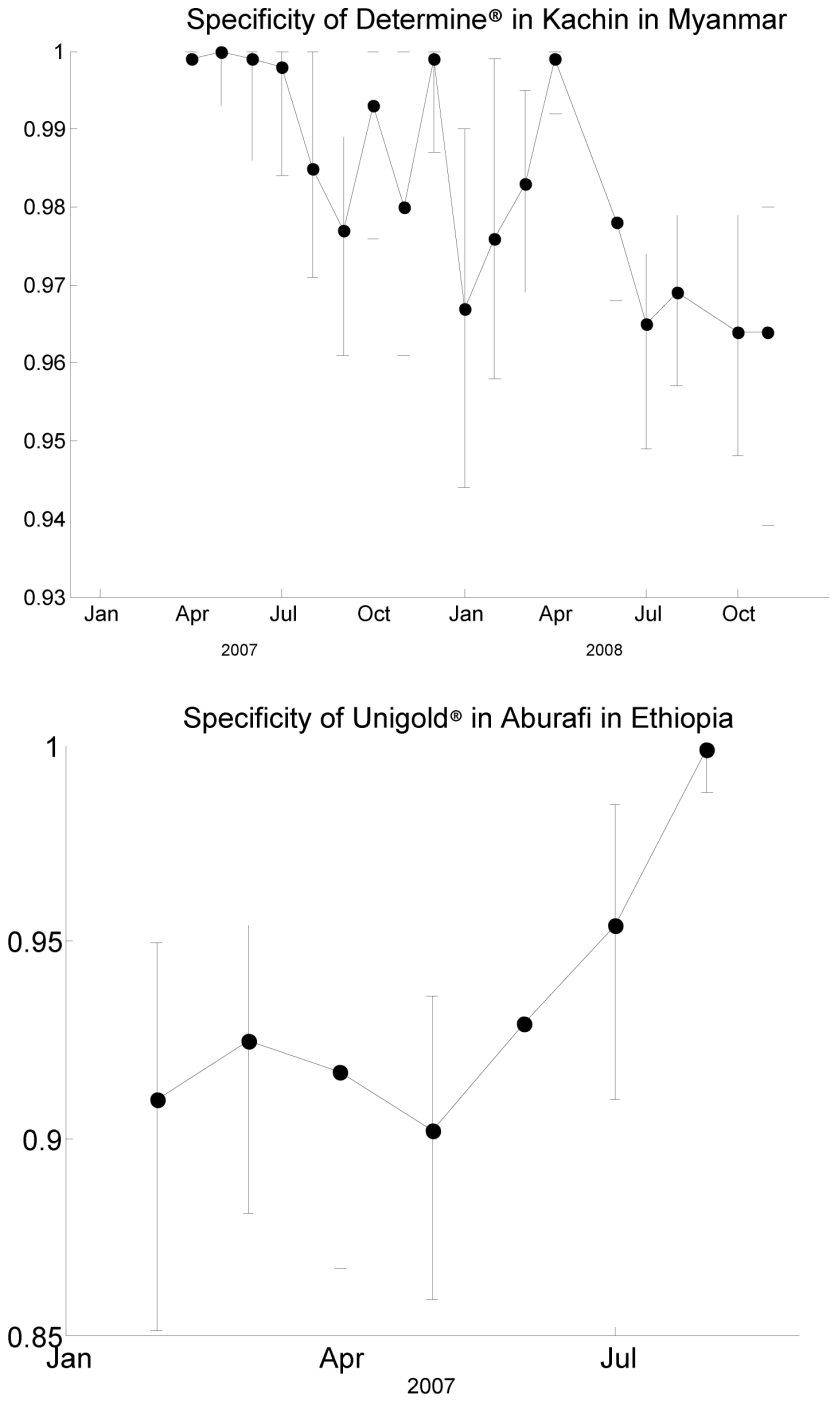
Estimates of specificity of Determine® in Kachin, Myanmar and Unigold® in Abdurafi, Ethiopia.

We demonstrate variation in individual test specificity over time using a site in Myanmar and another in Ethiopia as examples. By focusing on individual sites, rather than pooled estimates over an entire country, we avoid any artificial effects resulting from varying prevalence between sites. The model also includes independent estimates of prevalence at each time point, to adjust for any changes in prevalence over the time period. Figure 4 presents estimates and 95% credible intervals for specificity of Determine® in Kachin in Myanmar, and for specificity of Unigold® in Abdurafi in Ethiopia. These two examples show clear evidence of changing specificity over time within a single testing site, with one test becoming more specific and the other less specific.

While these two programmes are not the only ones to show temporal changes in specificity, it should be noted that not all tests sites showed evidence of variation over time: some did not have sufficient months of testing with the same test to explore temporal changes, while others showed consistency in test characteristics over time. The examples in Figure 4 have been deliberately chosen to highlight the existence of variation over time that is not exclusive to a single test, and is not either a consistent improvement or decline in specificity.

### Role of test sensitivity

The results presented here focus on changes in test specificity by place and over time. To investigate the role of test sensitivity in discordant results, we calculated the expected proportion of discordant results due to false negatives. Over all locations and months, we estimated that 9.9% of discordant results were due to false negatives, with the remaining 90.1% due to false positives. Countries with the highest proportion of false negatives were India (38.4%) and Congo-Brazzaville (32.2%). In India, these false negatives were largely driven by low estimated sensitivity of Tridot®, while in Congo-Brazzaville, sensitivity of Unigold® was estimated to be low. However, both these estimates were made from small numbers of samples: estimates in Congo-Brazzaville were made from only 10-50 monthly HIV tests, while uncertainty in estimates of test sensitivity for Tridot® arose from data for a single country with relatively small numbers of discordant cases. Complete results of the sensitivity analysis by country and test site are available as Supporting Information (Figure S4). 

 As our best-fit model showed high sensitivity in almost all locations, we also considered estimates from an additional model that assumed perfect sensitivity of all tests. Using this perfect sensitivity model, the estimates of prevalence and test specificity were only slightly changed from the best fit model. Almost all (around 96%) of the specificity estimates were within 1% of our previous estimates, with most of the big changes due to small numbers of tests in Congo-Brazzaville. Our main findings are unchanged in this model: our example sites in Figure 4 still show clear variation in specificity over time, and there are more sites indicating changes in test specificity over time in this model than the best fit model. Further details on these analyses are provided as Supporting Information (see Annexe S1). 

## Discussion

The results demonstrate three key points regarding HIV RDTs. Firstly, specificity varies by location, both within and between countries. Secondly, specificity varies over time within a particular location and thirdly these variations are not confined to a single test.

Possible explanations for the variability in the discordancy patterns seen in our data are changes in test performance (i.e. sensitivity or specificity) or quality of testing. We have controlled for differences due to quality of testing and for test device failure by repeating all results in the laboratory, and through a systematic quality control system. Changes in sensitivity, either due to variations in the test’s ability to detect early seroconversion or due to genetic HIV viral diversity are other potential causes for discordancy. Our modelling however allowed us to look at the effect of specificity while controlling for changes in sensitivity and prevalence of HIV. Within the model, both time and geographic location were important for explaining these data. Sensitivity for most tests was good, with the exception of Unigold® which showed considerable variation and Tridot® in India. A comparison of our results with a model assuming perfect sensitivity showed an adequate fit to the data, and left our estimates of prevalence and test specificity only marginally changed from the best-fit model. Given the high estimates of sensitivity for most tests in most countries, and the adequate fit of a model excluding variation in sensitivity, we conclude that variation in test sensitivity plays little role in explaining changes in discordant tests.  By allowing for variable sensitivity in our full model, we have adjusted for any small changes in discordancy patterns that could be attributable to changing sensitivity.

We postulate that cross reactive antibodies are the most likely cause of the variability in specificity in the MSF programmes described in this paper. Cross reacting antibodies react variously and unpredictably which is consistent with the observed temporal changes within individual programmes, and are likely to vary in prevalence between different population groups [10,11]. Cross reactivity with non-specific polyclonal B-lymphocyte antibodies formed in the early immune response has been reported as a cause of HIV testing interference [12–14] and this activity may be heightened in the resource-limited settings in which MSF operates[14–16].

Geographic differences in the performance of HIV RDTs have been previously documented, particularly for the Sub-Saharan Africa region. The varying specificity has been most commonly attributed to cross reactivity due to exposure to infectious agents unique to the region such as malaria, schistosomiasis, or helminth infections [17,18]. Further investigations to determine if specific infections are more likely to cross-react with HIV serological tests, either directly or indirectly, have produced mixed results in identifying a single agent as responsible [12,19–22]. It is possible that variable antigenic exposure may explain some of both the geographic and temporal changes we found in our study, due to differing geographic or seasonal prevalence of an infectious agent or agents. We were unable to demonstrate a seasonal change over time due to lack of precise information on seasonality for all test centres and insufficient longitudinal data. Similarly due to lack of data on co-infections we were unable to draw any links between disease exposure at different test sites and our findings of varying specificity. 

Interestingly, researchers in Tanzania report evidence of geographic variability in specificity of the Determine® test but not Statpak® in the South West region of the country. They found an increased false positivity rate in low altitude regions but were unable to link this with specific infections such as malaria or schistosomiasis. They speculate that humoral immune responses against infections known to be more prevalent in lower altitudes could be responsible for their results [20]. 

A further possibility that may explain why cross reactivity would vary over time and place is that of population movements due to migration or displacement, both of which are common in resource limited settings. Displaced persons may have an increased vulnerability to infections such as malaria or schistosomiasis in a new location and may be in poorer health than residents. 

The result of our analysis suggest that the current guidelines and practice of selecting a national testing algorithm based on evaluations performed at a single point of time or in a single population are inadequate. While an individual test may meet the WHO recommended threshold for specificity of at least 98% at a single location or point in time [4], our results indicate it may not be possible to be extrapolate these results to other sites within the same country or region, nor can they be considered to be consistent over time. Given that even minor changes in specificity, particularly in low prevalence contexts, can lead to unacceptable positive predictive values, this has major implications for policy makers and patients undergoing HIV testing. There are important programmatic costs associated to falsely including someone in an HIV programme, in addition to the obvious individual costs of being falsely diagnosed [23]. 

Given this, we recommend that policy-makers re-examine the current guidance for determining and monitoring a test algorithm. A change would be needed that involves evaluation of algorithm performance against a gold standard at each geographic test site, and regular evaluation over time of test performance against the gold standard. An alternative approach which is more straightforward to implement given the serious practical challenges in implementing such a policy, could be to include confirmation testing for all patients as is done routinely in resource-rich settings. Incorporating confirmation testing into the algorithm would protect against the varying and unpredictable specificity we found in the test sites, and allow timely transmission of accurate results to clients. In our experience in these settings confirmation testing in the laboratory is both feasible and affordable [23,24]. 

Limitations to the review include the fact that it is routinely collected programmatic data and therefore subject to some gaps due to missing data. Another limitation is a bias introduced by the fact that high discordancy rates were responded to programmatically by either changing the choice of tests or moving to serial from parallel testing. This limits the range of specificity change profiled in this analysis. As the data used in the analysis is derived from discordant test results, our model is likely to under-estimate the rate of false positivity by excluding those samples whereby both tests are false positive. Double false positives, whereby both tests display poor specificity are well documented [17,24].. Finally our ability to measure variability over time was limited by the short period of data collection in some sites which may have underestimated the degree of variation. 

## Conclusion

This analysis demonstrates the variability of specificity of multiple independent HIV RDTs over time and geographic location. These results suggest a higher than previously appreciated risk of false positive HIV results using the current WHO guidelines for testing and evaluating diagnostic algorithms. This suggests a need to re-consider the current guidance for choosing and monitoring HIV testing algorithm performance in order to avoid the very significant personal and programmatic risk of false positive HIV results. A practical way to do this would be to incorporate a confirmation test into the test algorithm. 

## Supporting Information

Annexe S1 **Details of the Statistical Model**
(DOC)

Annexe S2 **Number of tests per test site**
(DOCX)

Figure S1 **Full model specification in WinBUGS**
(TIF)

Figure S2 **HIV prevalence by test site within each country with 95% credible intervals.**
(TIF)

Figure S3 **Specificity of each of the HIV tests, by country and test site with 95% credible intervals.** To allow for comparisons between countries, we have adopted the same vertical scale for each plot, although credible intervals for Ethiopia and Myanmar extend below 90% specificity in some sites.
(TIF)

Figure S4 **Sensitivity of each of the HIV tests, by country and test site with 95% credible intervals.** To allow for comparisons between countries, we have adopted the same vertical scale for each plot, although credible intervals for Ethiopia and India extend below 90% sensitivity in some sites.
(TIF)
