# Supplementary material for: Variation in Specificity of HIV Rapid Diagnostic Tests over Place and Time: An Analysis of Discordancy Data Using a Bayesian Approach
Source: PLoS One. 2013 Nov 25;8(11):e81656. doi: 10.1371/journal.pone.0081656 (PMC3840056; doi:10.1371/journal.pone.0081656)
Supplement: Annexe S1 — Details of the Statistical Model (DOC) [file pone.0081656.s001.doc]

**Annexe S1: Details of the Statistical Model**

We provide additional data on the statistical model used to estimate prevalence, specificity and sensitivity from the data, include additional graphs of estimated parameters, and report on our validation of both the temporal and non-temporal models.

For each location and month, there are five parameters to be estimated, namely:

- prevalence;
- specificity of each test (f_A_, f_B_);
- sensitivity of each test (v_A_, v_B_).

In order to estimate these parameters from the data, we need to define the probability of each of the test results in terms of the parameters, as:

Probability (positive-positive) = prevalence*v_A_*v_B_ + (1-prevalence)*(1-f_A_)*(1-f_B_)
Probability (positive-negative) = prevalence*v_A_*(1 – v_B_) + (1-prevalence)*(1-f_A_)*f_B_Probability (negative-positive) = prevalence*(1-v_A_)* v_B_ + (1-prevalence)*f_A_*(1-f_B_)

We can then relate these parameters to the data by assuming the data is sampled from a binomial distribution Bin (N, p), where N is the sample size, and p the probability of the event occurring, as:

number of positive-positives ~ Bin(N, Probability(positive-positive))
number of positive-negatives ~ Bin(N, Probability(positive-negative))
number of negative-positives ~ Bin(N, Probability(negative-positive))

For each site and month, we have four distinct data points, but five parameters to estimate, so we need to make further assumptions to ensure the model is well defined. We tested a number of different possible assumptions, including fixed specificity or sensitivity by location, a fixed ratio of sensitivity to specificity by location, and a fixed ratio of sensitivity to specificity by test. Over these assumptions, the following relationship was found to give the best fit to data, both in the full model, and in a simpler model that aggregated data over time:

v [location, test, month] = α [test] * f[location, test, month],

where α is a test-specific parameter.

Given this assumption, the model can be fitted to the data using Bayesian methods with the software WinBUGS. The full model specification is provided in Figure A1. To supplement the figures produced in the main text, we also provide estimates of prevalence, specificity, and sensitivity for all sites in the non-temporal model (see Figures A2-4). In particular, these figures show the variability in site prevalence and the sensitivity results by test.

In addition to measures of model fit provided by WinBUGS which allow us to distinguish between models, we tested the overall goodness of fit using simulation methods. Firstly, we generated synthetic data with known parameters for Model 2 (which includes variation by location but not by time), and re-estimated the test specificities. All estimates of specificity were within 1% of the true value. Secondly, we generated synthetic data based on our estimated parameters for Model 3 (which includes variation in both time and place), and compared this synthetic data to the true data. The median number of people with two positive tests in the synthetic data was within 1 of the true data in 95% of locations, and within 2 of the true value in over 99% of locations. Similar – or better agreement was found for the number of people with each type of discordant test result. These findings confirm that the models provide sufficient flexibility to reproduce the patterns seen in the data.

Finally, we used the full model (Model 3) including variation by place and over time to estimate the relative contribution of false positives and false negatives to discordant results. For each site and each month, we used our estimates of sensitivity, specificity and prevalence to estimate the number of discordant results due to false positives and the number of discordant results due to false negatives. Over all sites and all months, we estimated that 90.1% of discordant results were due to false positives. This proportion varied by country, with the lowest estimates for India (61.6%) and Congo-Brazzaville (67.8%). In India, false negatives were largely driven by low estimates of Tridot® sensitivity, while In Congo-Brazzaville, false negatives were produced by low sensitivity of Unigold®, as seen in Model 2. Both these estimates should be viewed with some caution as they reflect small numbers of tests in each country. In each of the other countries, the percentage of discordant results due to false positives was over 80%, with a number of countries having nearly all discordant results due to false positives.

As described in the paper, , we explored a comparison model assuming perfect sensitivity of all tests (that is v_A_ = v_B_ = 1). As is clear from the earlier equations, this assumption simplifies the model equations such that estimates of test specificity and disease prevalence could be calculated algebraically. Estimation of these parameters within WinBUGS allowed us to compare estimates of fit, providing a Deviance Information Criterion for this model of 6180. A comparison with estimates for other models shown in Table 2 shows an adequate fit, although less good than the reported best-fit model. A comparison of estimates of test specificity and disease prevalence in this perfect sensitivity model with our best-fit model showed good correspondence in almost all sites, with greatest differences in point estimates in those sites with small numbers of tests (such as Congo-Brazzaville). Using non-overlapping 95% credible intervals for specificity in different months in the same site as an indication of potential variation in specificity over time, we identified more sites indicating changes in specificity over time under the perfect sensitivity model than in the best-fit model. We conclude from this that the best fit model is attributing a small amount of the variation in discordancy patterns over time to sensitivity, and so producing conservative estimates of the amount of variation in specificity over time.
