# Supplementary material for: Variation in Specificity of HIV Rapid Diagnostic Tests over Place and Time: An Analysis of Discordancy Data Using a Bayesian Approach
Source: PLoS One. 2013 Nov 25;8(11):e81656. doi: 10.1371/journal.pone.0081656 (PMC3840056; doi:10.1371/journal.pone.0081656)
Supplement: Annexe S2 — Number of tests per test site (DOCX) [file pone.0081656.s002.docx]

| Country | Site number | Determine^®^ | Unigold^®^ | Capillus^®^ | Tridot^®^ | Hexagon^®^ | First Response^®^ | SD Bioline^®^ |
| --- | --- | --- | --- | --- | --- | --- | --- | --- |
| CAR | 1 | 4329 | 4329 |  |  |  |  |  |
| Congo-B | 1 | 438 | 438 |  |  |  |  |  |
| DRC | 1 | 1496 | 1496 |  |  |  |  |  |
|  | 2 | 3434 | 3434 |  |  |  |  |  |
|  | 3 | 2089 | 2089 |  |  |  |  |  |
|  | 4 | 396 | 396 |  |  |  |  |  |
|  | 5 | 553 | 553 |  |  |  |  |  |
|  | 6 | 1397 | 1397 |  |  |  |  |  |
|  | 7 | 3239 | 3239 |  |  |  |  |  |
|  | 8 | 5031 | 5031 |  |  |  |  |  |
|  | 9 | 1369 | 1369 |  |  |  |  |  |
|  | 10 | 61 | 61 |  |  |  |  |  |
| Ethiopia | 1 | 439 | 439 |  |  |  |  |  |
|  | 2 | 729 | 729 |  |  |  |  |  |
|  | 3 | 1901 | 1901 |  |  |  |  |  |
|  | 4 | 572 | 572 |  |  |  |  |  |
|  | 5 | 101 | 101 |  |  |  |  |  |
|  | 6 | 1219 | 1219 |  |  |  |  |  |
| Haiti | 1 | 6464 | 2006 | 4458 |  |  |  |  |
| India | 1 | 823 | 186 |  | 637 |  |  |  |
|  | 2 | 384 | 51 |  | 333 |  |  |  |
|  | 3 | 3972 | 887 |  | 3085 |  |  |  |
|  | 4 | 280 | 69 |  | 211 |  |  |  |
| Ivory Coast | 1 | 2652 | 1176 |  |  | 1476 |  |  |
|  | 2 | 2832 | 1063 |  |  | 1769 |  |  |
|  | 3 | 3550 | 1147 |  |  | 2403 |  |  |
| Myanmar | 1 | 15094 | 4214 | 10880 |  |  |  |  |
|  | 2 | 1376 |  | 1376 |  |  |  |  |
|  | 3 | 1441 |  | 1441 |  |  |  |  |
|  | 4 | 891 |  | 891 |  |  |  |  |
|  | 5 | 1358 | 391 | 967 |  |  |  |  |
|  | 6 | 2445 |  | 2445 |  |  |  |  |
|  | 7 | 841 | 569 | 272 |  |  |  |  |
|  | 8 | 629 | 422 | 207 |  |  |  |  |
|  | 9 | 762 | 445 | 317 |  |  |  |  |
|  | 10 | 1093 | 691 | 402 |  |  |  |  |
|  | 11 | 193 | 193 |  |  |  |  |  |
|  | 12 | 2196 | 867 | 1329 |  |  |  |  |
|  | 13 | 11501 | 7004 | 4497 |  |  |  |  |
| Uganda | 1 | 852 | 852 |  |  |  |  |  |
|  | 2 | 2801 | 2801 |  |  |  |  |  |
|  | 3 | 1066 | 1066 |  |  |  |  |  |
|  | 4 | 914 | 914 |  |  |  |  |  |
|  | 5 | 423 | 423 |  |  |  |  |  |
| Zimbabwe | 1 | 1351 |  |  |  |  | 831 | 520 |
|  | 2 | 1629 |  |  |  |  | 1221 | 408 |
|  | 3 | 1863 |  |  |  |  | 1535 | 328 |
|  | 4 | 952 |  |  |  |  | 221 | 731 |
|  | 5 | 1323 |  |  |  |  | 1323 |  |
|  | 6 | 2014 |  |  |  |  | 1703 | 311 |
|  | 7 | 1277 |  |  |  |  | 948 | 329 |
