# Supplementary figures and images for: Variation in Specificity of HIV Rapid Diagnostic Tests over Place and Time: An Analysis of Discordancy Data Using a Bayesian Approach
Source: PLoS One. 2013 Nov 25;8(11):e81656. doi: 10.1371/journal.pone.0081656 (PMC3840056; doi:10.1371/journal.pone.0081656)

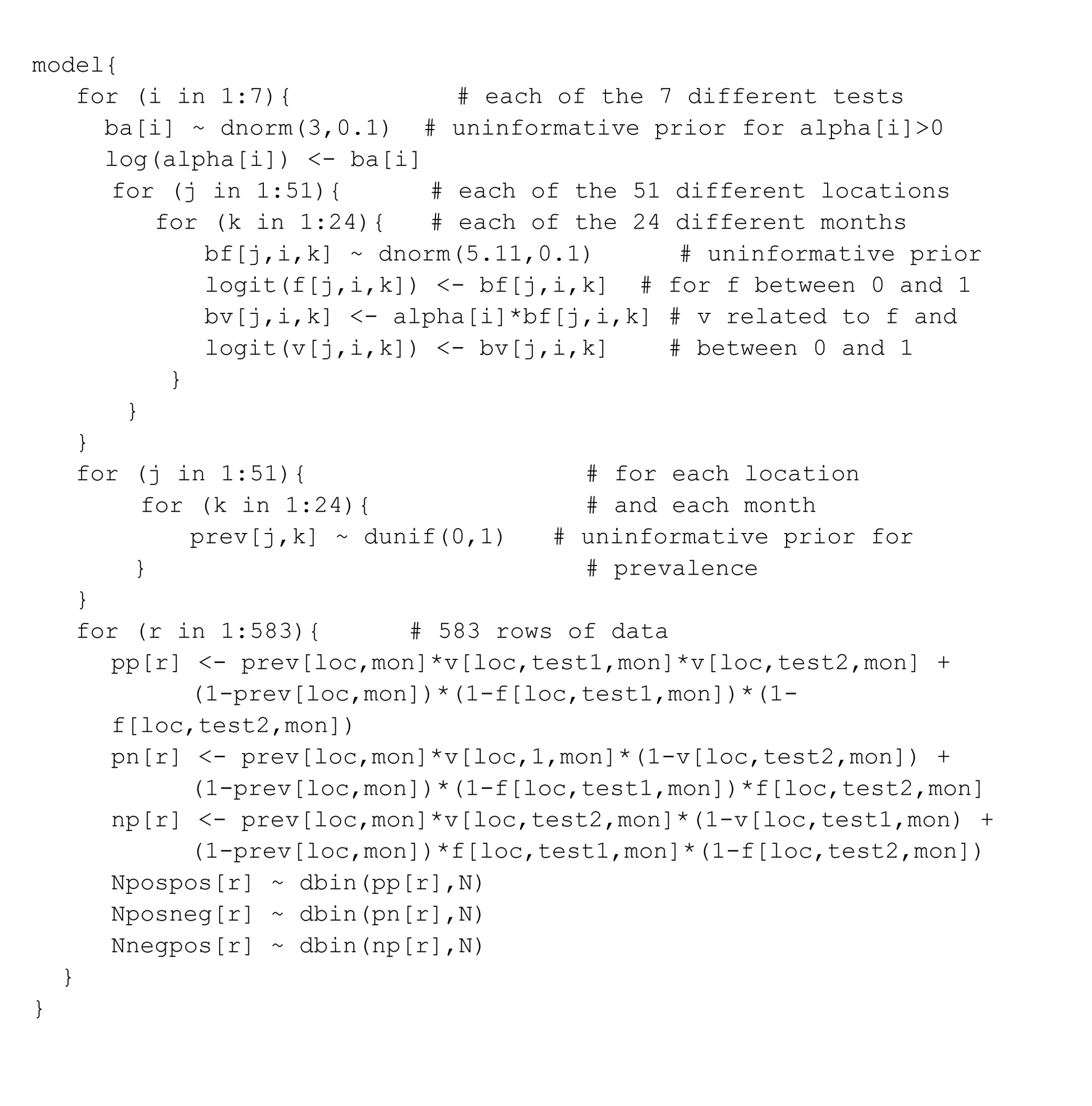

Supplement: Figure S1 — Full model specification in WinBUGS (TIF) [file pone.0081656.s003.tif]

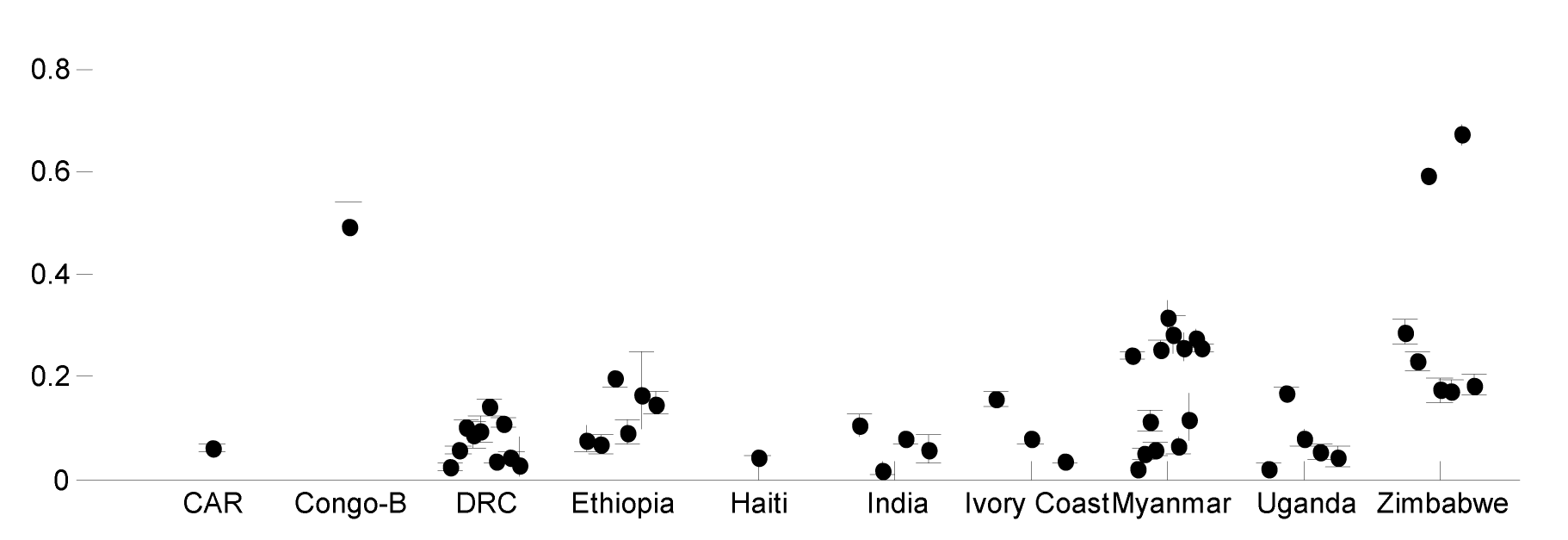

Supplement: Figure S2 — HIV prevalence by test site within each country with 95% credible intervals. (TIF) [file pone.0081656.s004.tif]

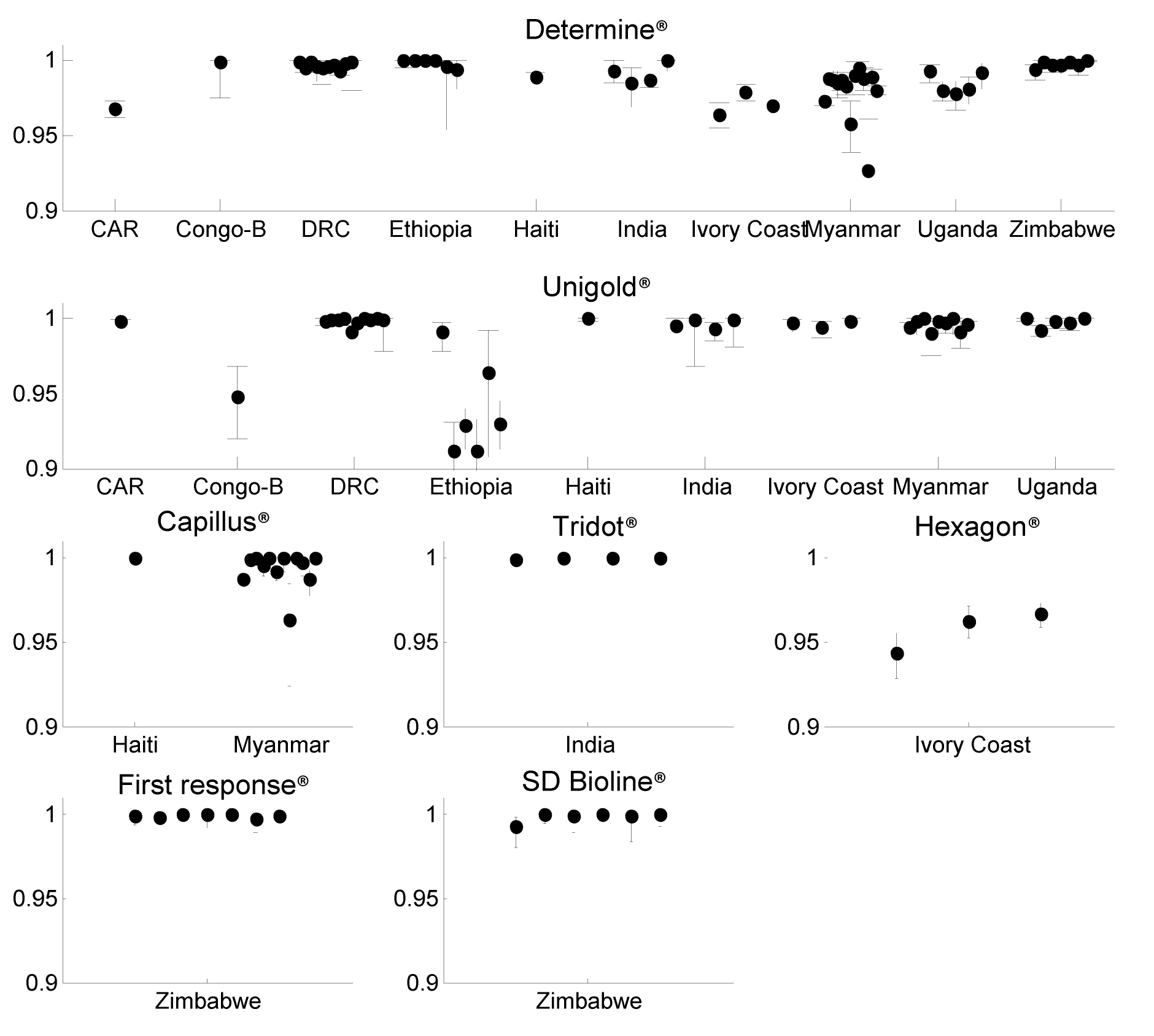

Supplement: Figure S3 — Specificity of each of the HIV tests, by country and test site with 95% credible intervals. To allow for comparisons between countries, we have adopted the same vertical scale for each plot, although credible intervals for Ethiopia and Myanmar extend below 90% specificity in some sites. (TIF) [file pone.0081656.s005.tif]

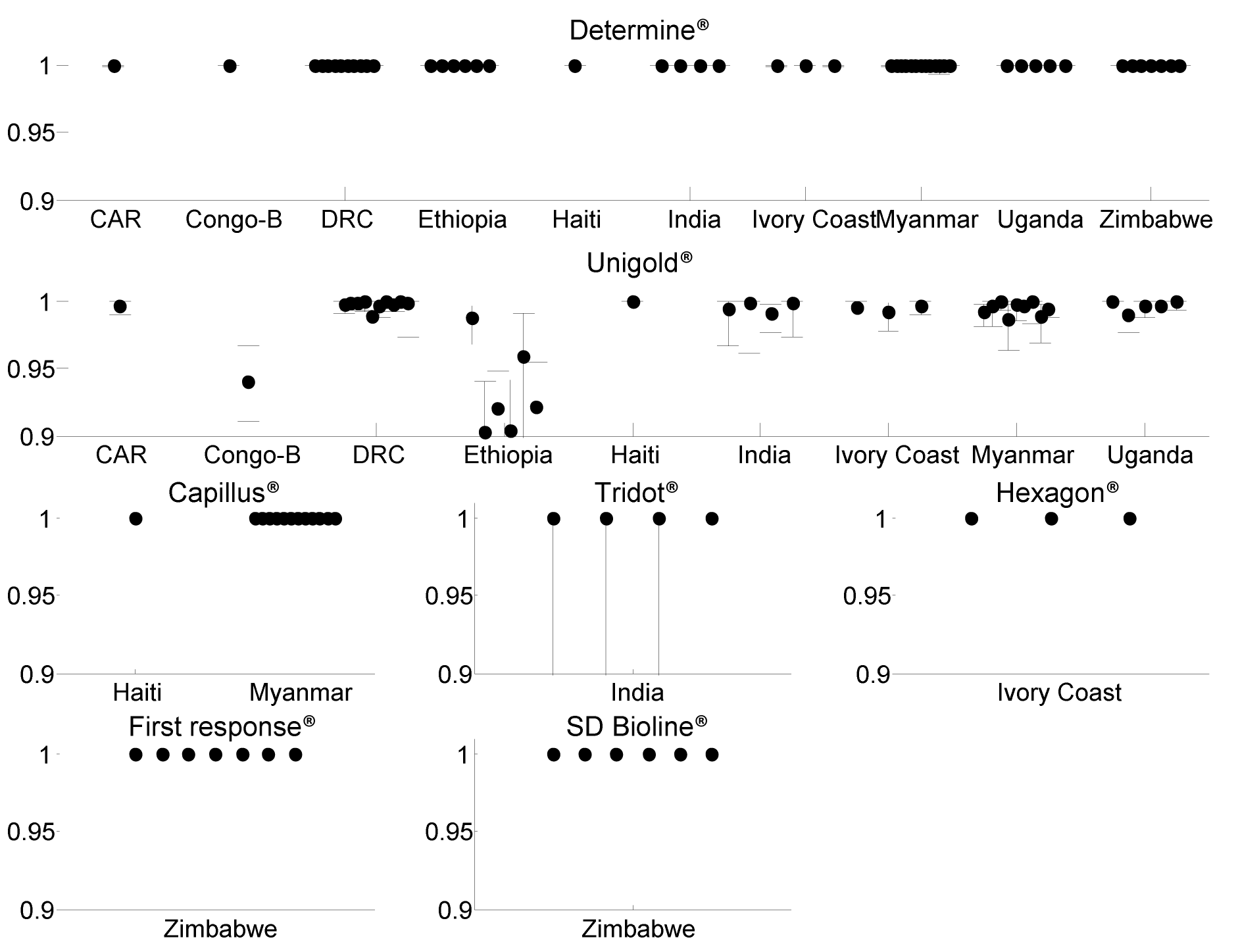

Supplement: Figure S4 — Sensitivity of each of the HIV tests, by country and test site with 95% credible intervals. To allow for comparisons between countries, we have adopted the same vertical scale for each plot, although credible intervals for Ethiopia and India extend below 90% sensitivity in some sites. (TIF) [file pone.0081656.s006.tif]
